# Assessment of the Optimal Distal End Length of Echogenic Perineural Catheters to Facilitate Successful Coiling: A Regional Anesthesia Simulator-Based Technical Study

**DOI:** 10.7759/cureus.101365

**Published:** 2026-01-12

**Authors:** Maria Riga, Eleni Poulogiannopoulou, Konstantina Panagouli, Erond Zeneli, Thomas Papadimos, Theodosis Saranteas

**Affiliations:** 1 Second Department of Anesthesiology, National and Kapodistrian University of Athens School of Medicine, Athens, GRC; 2 Department of Anesthesiology, The University of Toledo College of Medicine and Life Sciences, Toledo, USA

**Keywords:** coiling, perineural catheters, sciatic nerve, simulation, ultrasound

## Abstract

Introduction: We investigated the optimal length of a flexible end-portion of an echogenic-straight perineural catheter that would facilitate a catheter’s flexible distal portion to coil behind the sciatic nerve in a Blue Phantom nerve block simulator (Elevate Healthcare, Sarasota, Florida, United States).

Methods: In 80 20G perineural echogenic catheters, the integral stylet was retracted at various lengths (from 3 cm to 10 cm) so that the optimal catheter’s flexible length that enables the catheter’s flexible end portion (3-10 cm) to coil could be evaluated. Ten perineural catheters were allocated per cm of retraction. In our binary format, two variables were included: the first was the length of the catheter’s distal flexible end, and the second was whether the catheter’s distal end was coiled or not coiled. Optimal probabilities (90% and 95%) predicting the catheter’s optimal flexible distal length that facilitates successful coiling or not were evaluated by probit regression.

Results: Overall, 64 of the 80 perineural catheters were coiled. Analysis showed that withdrawing the integral stylet of a perineural catheter by 7 (6.1-9.4) cm and 8.1 (6.9-11.3) cm resulted in an optimal successful coiling with 90% and 95% probability (*p*<0.001). Injection of normal saline through the 60 unobstructed catheters contacted with the sciatic nerve in 55 cases (92%).

Conclusion: Retracting the integral stylet of a perineural catheter by specific lengths facilitates the coiling of its flexible distal end with high probability. This configuration could provide a functional buffer of catheter length, mitigating the risk of tip dislocation from its intended position.

## Introduction

A continuous peripheral nerve block (CPNB) is a point-of-care ultrasound technique of pain management (especially for upper and lower extremity injuries) that uses a catheter to continuously infuse a local anesthetic adjacent to a nerve structure, providing protracted periods of anesthesia and/or analgesia. Contextualizing CPNBs within the spectrum of multimodal analgesia, they are considered a technique that also offers an individualized, targeted, and site-specific pain remedy [1-3].

Notably, CPNB displacement can occur after placement because of various factors, including patient movement and/or inadequate fixation. This displacement results in catheter failure and insufficient analgesia, thus reducing postoperative care at the bedside [3-5]. However, a standard methodology for catheter advancement and placement, which could limit catheter displacement from its initial position during the postoperative period, is still lacking.

Of note, an echogenic catheter supported by an integral steel-stylet, but with a fixed distal flexible end-portion, is a catheter design that can be coiled adjacent to nerves, thereby providing an additional length of safety to mitigate potential catheter tip dislocation from its initial position [3-5]. Good clinical outcomes have also been reported using a flexible length of 6 cm, because it limits catheter displacement from its initial position [5]. However, studies that address the optimal length of a catheter’s flexible end that would facilitate a coiling maneuver in close proximity to nerves have not yet been designed.

The assessment of a novel technique ideally should take place in clinical trials. However, exposing patients to a new methodology may raise ethical issues. For this reason, studies in simulators allow assessment of a new technique in a safe/controlled experimental setting [6]. Therefore, we investigated the optimal length of a flexible (unsupported by stylet) end-portion of an echogenic straight perineural catheter that would facilitate a catheter’s distal end to coil behind the sciatic nerve on the first attempt, in a Blue Phantom regional anesthesia simulator (Elevate Healthcare, Sarasota, Florida, United States).

## Materials and methods

This was a technical simulator-based study that took place at the regional anesthesia simulation room of the Anesthesia Department of Attikon University Hospital of Athens, Greece. It was approved by the Ethics Committee of Attikon University Hospital (approval number: 11-13/11/2024).

Study procedure

**Figure 1:**
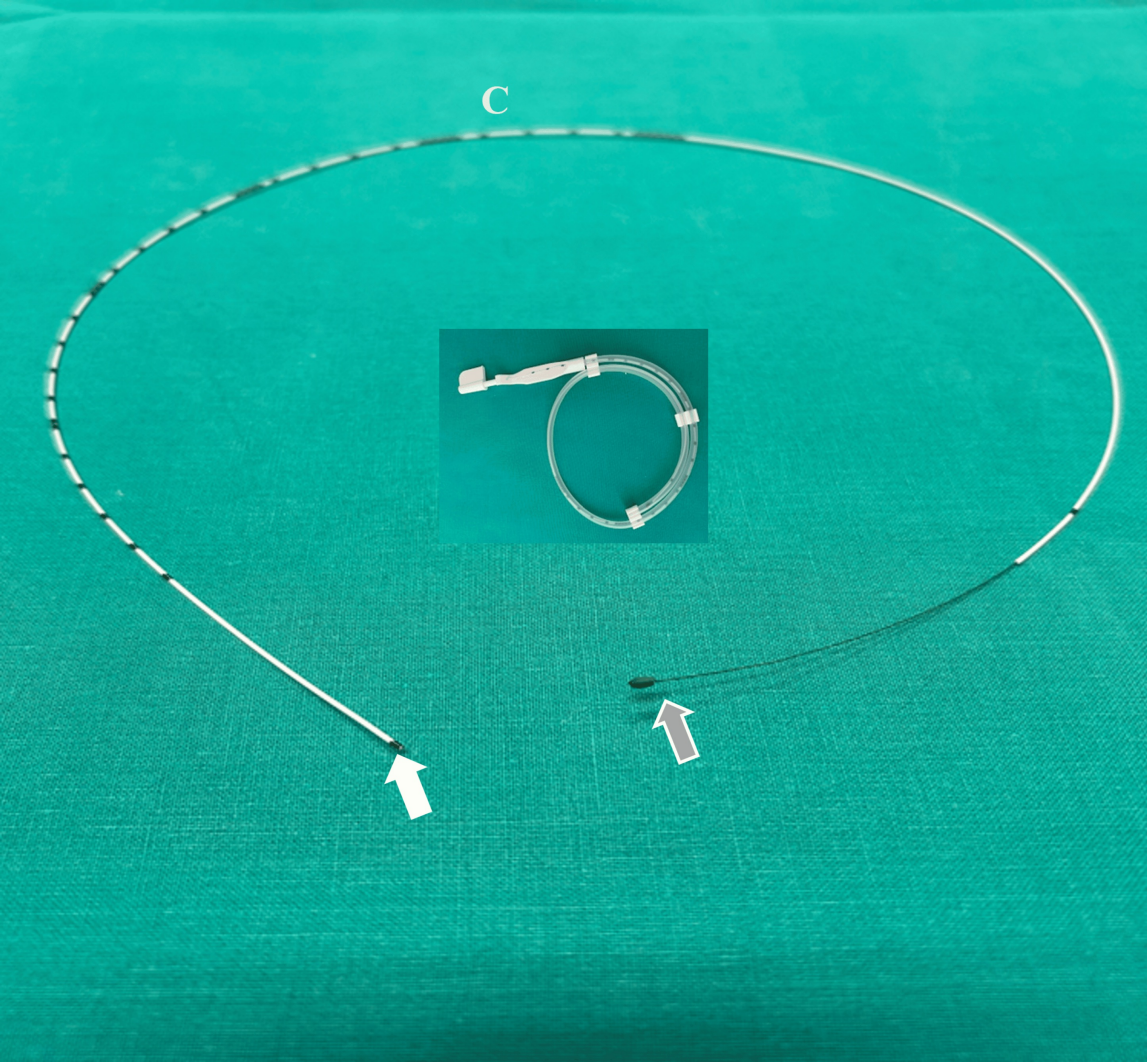
Straight perineural echogenic catheter with its integral stylet retracted The catheter’s integral stylet is retracted (grey arrow) so that the catheter’s distal portion features a more flexible end (white arrow). C: perineural catheter

The study was conducted in a Blue Phantom nerve block ultrasound simulator consisting of a nerve structure mimicking the sciatic nerve. Straight 20G/50cm perineural catheters, polyurethane-based (SonoLong/Sono, PAJUNK, Geisingen, Germany), with integral stylets, were employed. The stylet was removed at various lengths so that the optimal length of the flexible (unsupported by the stylet) distal end, ranging from 3 cm to 10 cm, could be evaluated (Figure 1). Eighty perineural catheters were used, and eight sets of numbers according to flexible distal end lengths (3-10 cm) were randomly generated (www.random.org/sequences/).

**Figure 2:**
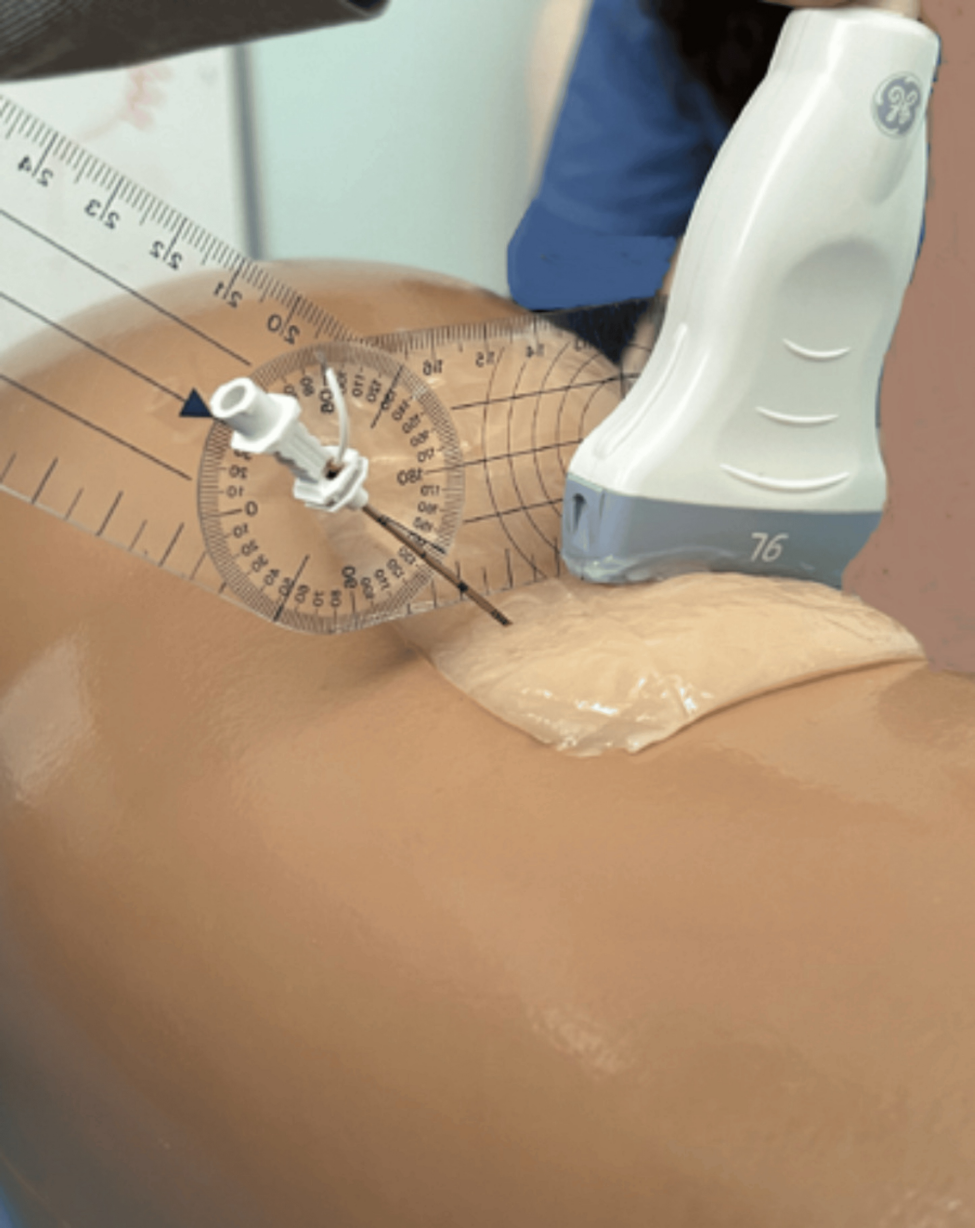
The needle insertion angle to the Blue Phantom posterior thigh surface as measured by goniometer

A Tuohy needle (18G × 7.5 cm; PAJUNK) was inserted (lateral-to-medial direction: needle in-plane, nerve in short-axis technique) at the upper third of the posterior thigh of a sciatic nerve simulator, under ultrasound real‐time guidance (5-9 MHz linear transducer, LOGIQ e; GE Healthcare Technologies, Inc., Chicago, Illinois, United States). The needle insertion angle to the Blue Phantom surface (coronal plane) was kept constant at 40 degrees (Figure 2); the needle was always aimed exactly behind and at the lateral edge of the nerve, with the curved end facing the targeted nerve. Prior to insertion, the catheter stylet was partially retracted to a length corresponding to the pre-specified flexible distal segment. The perineural catheter (with stylet unsupported distal end length) was subsequently inserted through the needle and advanced in the medium according to the catheter’s flexible distal end length allocation, ensuring that only the unsupported portion dictated the catheter’s navigational dynamics. One attempt of 15 seconds was permitted for the catheter’s flexible end portion to coil underneath the sciatic nerve, as previously described [4].

**Figure 3:**
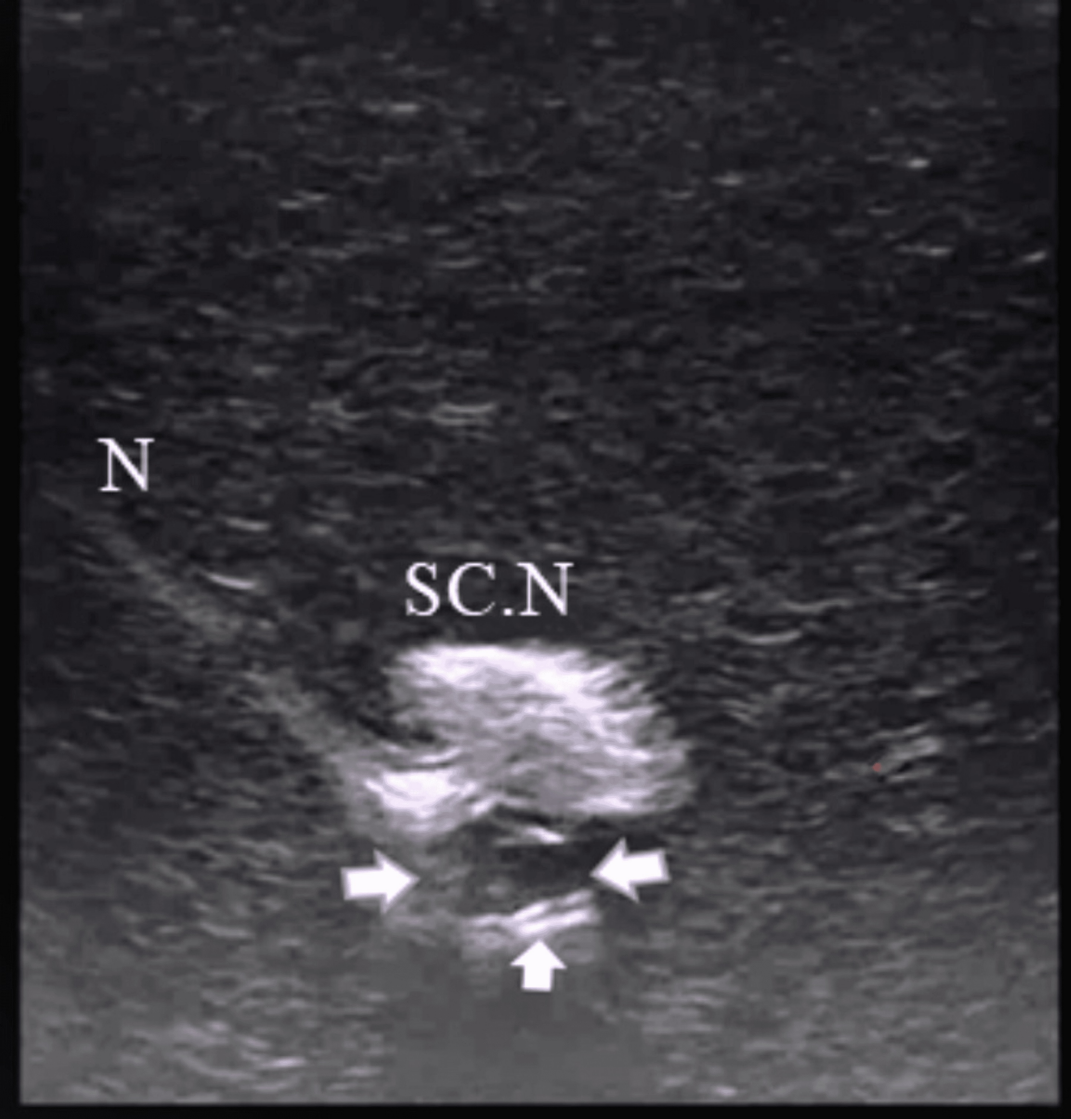
The coiling perineural catheter (arrows) develops a complete, closed distal end loop under the sciatic nerve of the Blue Phantom simulator SC.N: sciatic nerve; N: needle

A video loop of each catheter coiling maneuver (15 seconds) was obtained under the same imaging settings and stored for offline analysis (frame by frame) by two independent investigators who were blind to group allocation. The investigators had to decide whether the flexible distal end of each perineural catheter was successfully coiled. A perineural catheter was considered coiled if one or more complete catheter’s distal end loops were structured (the longer the stylet unsupported distal end length, the greater the number of loops formed by the catheter's flexible portion). All catheter’s distal end loops were clearly visualized after a careful frame-by-frame video analysis (Figure 3, Video 1). Cases in which the catheter’s distal end was bent and not coiled, or it bypassed the nerve and was coiled afterwards (but not behind the nerve), were considered failures. Normal saline (3 ml) was then injected through the catheter by the investigator performing the nerve block to confirm the catheter’s patency. The proportion of cases where normal saline spread in the testing medium came into contact with the sciatic nerve was assessed. The results were considered positive only after unanimous agreement between the two independent researchers.

**Video 1:** The perineural catheter coils underneath the Blue Phantom’s sciatic nerve insert.

Statistical analysis

In our binary format, two variables were included: (i) the length of the catheter’s distal flexible end (stylet unsupported by 3-10 cm), and (ii) whether the catheter’s distal flexible end was successfully coiled (1) or not coiled (0) behind the nerve. Hence, the optimal predicted probabilities (90% and 95%) of a positive response (coiled catheter) were evaluated by using probit regression. Interobserver agreement was assessed by using the inter-rater agreement statistic, Kappa. IBM SPSS Statistics for Windows, version 23.0 (IBM corp., Armonk, New York, United States) and MedCalc Software Ltd, Ostend, Belgium, were used for analysis.

## Results

Video analysis and perineural catheter assessment

The percentages of successful perineural catheter coiling and the characteristics of the coiled catheters relative to the allocated distal end length are presented in Table 1. Overall, 80 video loops were assessed. Of these, 64 (80%) perineural catheters were coiled behind the sciatic nerve. In four out of the 64 cases (6%), catheters were found to be coiled and obstructed; however, patency was restored following partial uncoiling. Nonetheless, these catheters were not included in further analysis (normal saline contact with the sciatic nerve). In 55 out of the remaining 60 instances (92%) involving coiled catheters, the injection of normal saline resulted in visible contact between the infusion and the targeted nerve. Upon removal, no catheter knotting was observed.

**Table 1:** Characteristics of the perineural catheters after placement per allocated flexible catheter distal end length.

Catheter flexible distal end length (stylet unsupported)	Successful coiling, n (%)	Obstruction of coiled catheters, n (%)	Contact of saline spread with the sciatic nerve, n (%)
3 cm	5/10 (50%)	1/5 (20%)	4/4 (100%)
4 cm	4/10 (40%)	0/4 (0%)	3/4 (75%)
5 cm	8/10 (80%)	0/8 (0%)	8/8 (100%)
6 cm	9/10 (90%)	1/9 (11.1%)	6/8 (75%)
7 cm	10/10 (100%)	0/10 (0%)	9/10 (90%)
8 cm	9/10 (90%)	1/9 (11.1%)	8/8 (100%)
9 cm	9/10 (90%)	0/9 (0%)	9/9(100%)
10 cm	10/10 (100%)	1/10 (10%)	8/9 (89%)

Probit regression analysis

**Figure 4:**
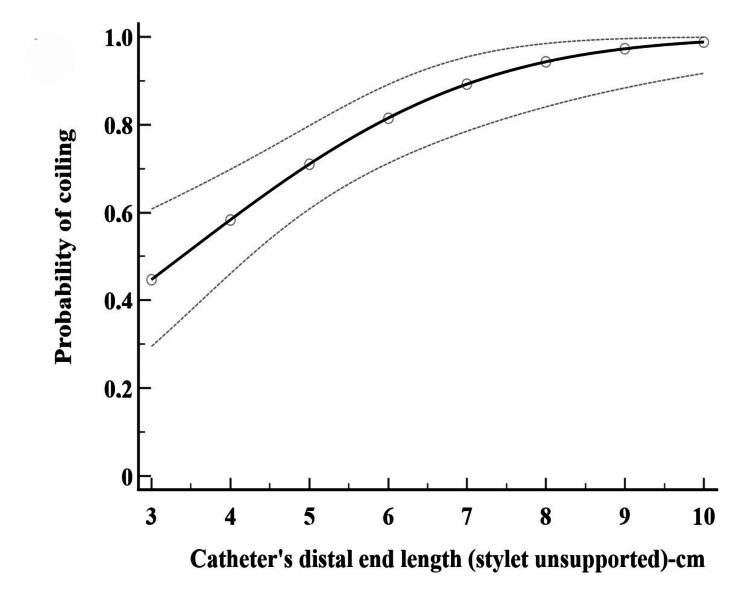
The relationship between the catheter’s distal flexible end length (stylet unsupported by 3-10 cm) and the catheter’s distal end coiling. The 95% probability is estimated by probit regression analysis

Probit analysis showed that withdrawing the catheter’s integral stylet by 7 (6.1-9.4) cm and 8.1 (6.9-11.3) cm resulted in an optimal successful coiling with 90% and 95% probability (p<0.001), respectively (Figure 4).

Interobserver variability

There was significant agreement between the two investigators assessing perineural catheter coiling behind the sciatic nerve and contact of normal saline spread with the sciatic nerve (kappa=0.95 and kappa=1, respectively).

## Discussion

In our investigation, the integral stylet was partially withdrawn to various lengths, balancing malleability and stiffness to enable the catheter’s distal segment to coil. From a technical perspective, the combination of the integral stylet and wire reinforcement ensured the catheter body remained rigid and kink-resistant; this allowed the flexible unsupported distal portion to bend and coil against tissue resistance upon exiting the curved tip of the Tuohy needle.

Previous research using a Blue Phantom sciatic nerve simulator demonstrated that a 6-cm stylet unsupported catheter exhibited superior coiling performance compared to a catheter supported by a stylet throughout its entire length. However, this outcome was not obtained upon the first attempt [4]. Therefore, the optimal balance of malleability and stiffness needed to achieve ease of the catheter’s coiling on the first attempt was investigated by retracting the integral stylet at different lengths.

Τhe probit analysis showed that a stylet retraction length of 8.1 cm may present an optimal condition in which the perineural catheter’s flexible distal end length could coil under the nerve structure on the first attempt with high probability. Moreover, in 92% of the coiled unobstructed catheters, the 3 ml normal saline injection spread came into contact with the nerve structure, thus confirming a very close placement of the perineural catheter tip underneath the sciatic nerve (Video 2). While 8% of catheters failed to achieve initial contact between the saline injectate and the sciatic nerve, this does not necessarily indicate unsuccessful placement. Rather, it reflects a measurable distance between the catheter tip and the nerve; this carries no clinical significance, given the larger volumes involved in clinical practice.

**Video 2:** Normal saline spread injected through the coiled catheter comes into contact with the Blue Phantom’s sciatic nerve insert. SC.N: sciatic nerve; *: normal saline; C: perineural catheter

A Tuohy needle was utilized because its curved tip directed the catheter’s end portion to a curving trajectory that facilitated a coiling maneuver behind the sciatic nerve. Conversely, straight-beveled needles typically direct perineural catheters along a linear path, which is not expected to produce an equivalent degree of curvilinear trajectory. We always calibrated needle-insertion angles at 40 degrees with the coronal plane to maintain constant angulations between the catheter’s tip and the surrounding Blue Phantom material, while the catheter was threaded through the Tuohy needle. We did not select a lower than 30 degrees angle, because it could enhance catheters’ visibility (due to low background echogenicity of the Blue Phantom medium), while a higher than 45 degrees angle could be very steep to assess the trajectory of a catheter [7,8]. In fact, the two investigators successfully reviewed the catheters’ coiling maneuver with significant interobserver agreement.

Notably, the relation between the distal flexible end length of perineural catheters and the corresponding success (coiled) or failure (not coiled) response has not yet been examined. In our research, a sigmoidal functional-response curve was utilized to analyze the flexibility-coiling relationship, characterizing how the balance between malleability and stiffness in the catheter’s distal segment directly influences the coiling response. Notably, a preexisting pertinent model describing this relationship is lacking. To address this, probit regression (a generalized linear model for binomial responses) was employed to provide a linear transformation of an observed sigmoidal functional-response curve [9-11]. This analysis was particularly suitable because successful catheter coiling maneuvers consistently followed a predictable progression: specifically, the probability of catheter coiling increased as the stylet was further retracted before almost reaching a plateau phase.

In our research, a non-sequential model and probit analysis were applied, which have previously been used in anesthesia research [9]. We did not use a sequential design (e.g., up and down methods), which would have included lower number of experiments in comparison with a non-sequential design, because our study did not involve humans (exposed to a new methodology) and our attempt was to better understand the performance of a catheter coiling manoeuvre by examining different catheter flexible distal end lengths under this methodology [9]. Hence, given that a non-sequential design requires twice as many cases to achieve the same lower mean systemic error as a sequential design (methodically including 20-40 cases) [10,11], we performed 80 experiments (distributed in eight groups) under a non-sequential methodology.

Our study has certain limitations. A shortcoming is that we conducted our research in a Blue Phantom simulator and not on biological tissues (i.e., cadavers). Nevertheless, Blue Phantom simulators have almost comparable mechanical characteristics to humans; more importantly, they exhibit significant durability as well as “self-healing” properties after multiple needle punctures and saline injections, thus providing significant repeatability of standard research conditions [6,12].

The Blue Phantom simulators are constructed from an elastomeric rubber that presents structural memory and high viscoelasticity properties, allowing the material to reconstitute when the needle is withdrawn or normal saline is injected. Specifically, injections of normal saline through the needle and/or the perineural catheter make the elastomeric medium expand locally. However, due to the material’s restorative elastic tension, the medium returns to its baseline configuration following the saline injection. This elastic recovery serves to expel infused saline through the primary needle insertion tract, thus preventing fluid accumulation [6,12]. Consequently, an injection of saline through the needle prior to catheter advancement (a standard technique in clinical practice) could not be performed. This fundamental difference from clinical practice significantly confines the clinical application and generalizability of the present findings. Nevertheless, the current study aimed to rigorously evaluate the potential interaction dynamics of this specific, polyurethane-based, perineural catheter within a substrate (engineered to provide high durability in conjunction with human-tissue-equivalent properties), thus analyzing the intrinsic ability of the catheter’s distal end to perform a coiling maneuver under standard research conditions.

Furthermore, although the catheter’s end length could move in a three-dimensional (3D) manner within the simulator material, we used a two-dimensional (2D) ultrasound image to assess the catheter’s coiling. While with 3D image and image reconstruction, the number and size of complete loops could be clearly evaluated, 2D ultrasound imaging (due to its higher spatial resolution than 3D imaging) could provide detailed visualization of a catheter’s motion, as well as sequences of video loop frames for thorough post-capture analysis [13]. Additionally, because the linear transducer's footprint was wider than that of the outer diameter of the perineural catheters (20G or 1.1 mm), it provided a sufficient field of view (with respect to the catheter’s outer diameter) that allowed clear depiction of the catheter’s motion in the testing medium. For this reason, stored video loops (Video 1) were examined offline multiple times by the two investigators, who precisely evaluated the course of the catheter within the testing medium, thus securing the possible coiling maneuver. In fact, the interobserver variability between the two independent investigators was negligible.

One technical shortcoming observed is the tendency for the distal end portion of coiled catheters to kink. Nevertheless, obstructed catheters occurred infrequently; this defect can be mitigated by partial uncoiling, a maneuver that successfully unblocks the site of obstruction

We did not consider a coiled distal end length greater than 10 cm due to the probability of catheter knotting [14], nor did we examine the coiling ability of a catheter with a flexible distal end length of less than 3 cm, because we hypothesized that such an end length would not successfully develop a closed (of sufficient length) distal end coiled loop. This decision was reasoned by the fact that a perineural catheter of 2.5 cm self-coiling distal end (currently used in clinical practice) features an open (incomplete) distal end coiled loop [15]. Despite their favorable clinical performance [15], head-to-head comparisons with flexible distal end portion catheters remain an area that has not been investigated.

## Conclusions

Our study suggests that partially retracting the integral stylet of a 20G straight echogenic perineural catheter by a specific length (8.1 cm) increases the likelihood of achieving a successful coiling maneuver. This technique provides an additional safety margin at the distal end of the catheter near the sciatic nerve, thereby mitigating the risk of tip dislocation in the event of accidental displacement.

This study generates hypotheses for clinical trials rather than defining practice. An 8.1 cm distal end length represents a probabilistic optimum rather than a prescriptive clinical target for successful coiling. Therefore, factors such as inter-patient anatomical variations, tissue compliance, and heterogeneity in perineural catheter designs, and CPNBs may influence the success of the coiling maneuver across various clinical settings; investigation through well-designed clinical trials is warranted to determine clinical utility.

## References

[1] Saranteas T, Koliantzaki I, Savvidou O, Tsoumpa M, Eustathiou G, Kontogeorgakos V, Souvatzoglou R (2019). Acute pain management in trauma: anatomy, ultrasound-guided peripheral nerve blocks and special considerations. Minerva Anestesiol.

[2] Drapeau-Zgoralski V, Bourget-Murray J, Hall B (2022). Surgeon-performed intraoperative peripheral nerve blocks and periarticular infiltration during total hip and knee arthroplasty: a critical analysis review. JBJS Rev.

[3] Luyet C, Meyer C, Herrmann G, Hatch GM, Ross S, Eichenberger U (2012). Placement of coiled catheters into the paravertebral space. Anaesthesia.

[4] Saranteas T, Poulogiannopoulou E, Riga M, Panagouli K, Mavrogenis A, Papadimos T (2024). Coiling of echogenic perineural catheters with integral stylet: A proof-of-concept randomized control trial in a sciatic nerve block simulator and a pilot study in orthopaedic-trauma patients. F1000Res.

[5] Saranteas T, Poulogiannopoulou E, Ntalamagka G, Skaligkou P, Giasafaki M, Papadimos T (2024). Perineural coiled echogenic catheters with a flexible distal end: A brief technical report. Anaesth Crit Care Pain Med.

[6] Hocking G, Hebard S, Mitchell CH (2011). A review of the benefits and pitfalls of phantoms in ultrasound-guided regional anesthesia. Reg Anesth Pain Med.

[7] Mariano ER, Yun RD, Kim TE, Carvalho B (2014). Application of echogenic technology for catheters used in ultrasound-guided continuous peripheral nerve blocks. J Ultrasound Med.

[8] Elsharkawy H, Maheshwari A, Farag E, Mariano ER, Rosenquist RW (2016). Development of technologies for placement of perineural catheters. J Anesth.

[9] Bouvet L, Da-Col X, Chassard D (2011). ED₅₀ and ED₉₅ of intrathecal levobupivacaine with opioids for Caesarean delivery. Br J Anaesth.

[10] Pace NL, Stylianou MP (2007). Advances in and limitations of up-and-down methodology: a précis of clinical use, study design, and dose estimation in anesthesia research. Anesthesiology.

[11] Stylianou M, Flournoy N (2002). Dose finding using the biased coin up-and-down design and isotonic regression. Biometrics.

[12] Chen FM, Liu X (2016). Advancing biomaterials of human origin for tissue engineering. Prog Polym Sci.

[13] Fenster A, Parraga G, Bax J (2011). Three-dimensional ultrasound scanning. Interface Focus.

[14] De Tran QH, De La Cuadra-Fontaine JC, Chan SY, Kovarik G, Asenjo JF, Finlayson R (2007). Coiling of stimulating perineural catheters. Anesthesiology.

[15] Nickl R, Vicent O, Müller T, Osmers A, Schubert K, Koch T, Richter T (2022). Impact of self-coiling catheters for continuous popliteal sciatic block on postoperative pain level and dislocation rate: a randomized controlled trial. BMC Anesthesiol.

